# Periodontal Disease and Endo-Periodontal Lesion Subtypes: A Retrospective Study

**DOI:** 10.3390/healthcare14162551

**Published:** 2026-08-14

**Authors:** Irina Bodnar, Anca Silvia Dumitriu, Stana Păunică, Marina Cristina Giurgiu, Fidan Bahtiar Ismail, Brindușa Florina Mocanu, Nicolae Dragoș Ciongaru, Ștefan Dimitrie Albu, Ioana Suciu, Roxana Eliss Budei

**Affiliations:** 1 Department of Periodontology, Faculty of Dental Medicine, Carol Davila University of Medicine and Pharmacy, 8 Dionisie Lupu Street, Sector 2, 020021 Bucharest, Romania; anca.dumitriu@umfcd.ro (A.S.D.); stana.paunica@umfcd.ro (S.P.); marina.giurgiu@umfcd.ro (M.C.G.); fidan.ismail@umfcd.ro (F.B.I.); brandusa.mocanu@umfcd.ro (B.F.M.); nicolae-dragos.ciongaru@drd.umfcd.ro (N.D.C.); stefan.albu@umfcd.ro (Ș.D.A.); 2 Department of Endodontics, Faculty of Dental Medicine, Carol Davila University of Medicine and Pharmacy, 8 Dionisie Lupu Street, Sector 2, 020021 Bucharest, Romania; ioana.suciu@umfcd.ro; 3 Department of Restorative Dentistry, Faculty of Dental Medicine, Carol Davila University of Medicine and Pharmacy, 8 Dionisie Lupu Street, Sector 2, 020021 Bucharest, Romania

**Keywords:** endo-periodontal lesions, periodontitis, diabetes mellitus

## Abstract

**Background/Objectives:** Endo-periodontal lesions (EPLs) represent complex pathological entities involving both periodontal and pulpal tissues, often implying diagnostic and therapeutic challenges. Limited evidence exists regarding the relationship between periodontal disease staging and grading and EPL subtype. The aim is to evaluate the clinical and statistical distribution of endo-periodontal lesions and investigate their association with periodontal stage and grade according to the European Federation of Periodontology (EFP) classification. **Methods:** An observational retrospective study was conducted in the Department of Periodontology, Faculty of Dental Medicine, Carol Davila University of Medicine and Pharmacy, Bucharest, Romania. Among 884 examined patients, 31 of them were diagnosed with EPLs and included in the analysis. Demographic variables, systemic conditions, affected tooth type, EFP stage and grade, and EPL type according to the Foce classification were recorded. Statistical analysis included Fisher’s exact test and univariable and multivariable binomial logistic regression models. **Results:** The mean age of the patients was 53.55 ± 9.42 years, with a nearly equal gender distribution. Most patients presented Stage III periodontitis (54.8%), Grade B periodontitis (71%), and EPL 3 (58.1%). Pluriradicular teeth were the most frequently affected (64.5%). The only statistically significant association identified was between EFP grading and EPL classification (*p* = 0.001). Grade B patients were significantly more likely to present EPL 3 than Grade C patients. Logistic regression analysis demonstrated that Grade B was a strong predictor of EPL 3 both in the univariable model (OR = 27.2; 95% CI: 2.712–272.828) and after adjustment for age and gender (OR = 32.676; 95% CI: 2.832–376.984). Neither age nor gender showed a significant association with EPL 3 occurrence. **Conclusions:** EPL 3 was the predominant lesion subtype in the study. EFP grading was significantly associated with EPL distribution, with Grade B emerging as an independent predictor of EPL 3. These findings suggest that periodontal disease progression may play a key role in the development and characterization of combined endo-periodontal lesions and highlight the importance of integrated periodontal–endodontic assessment in clinical practice.

## 1. Introduction

The periodontium and the pulpal space represent the two primary sites of dental infection of bacterial origin. Although anatomically separated, these tissues may communicate through the apical foramen, dentinal tubules, accessory canals, and root fractures, allowing bacterial spread and the development of inflammatory responses in both compartments.

In clinical practice, periodontists, endodontists, and general dental practitioners frequently collaborate to determine whether a lesion is of endodontic or periodontal origin or represents a true combined endo-periodontal lesion (EPL), requiring integrated endodontic and periodontal management. The diagnosis and treatment of EPLs remain challenging because of their multifactorial etiology, overlapping clinical presentation, and variable prognosis. Treatment outcomes largely depend on accurate identification of the primary source of infection and the extent of periodontal destruction [1,2,3].

The first widely used classification of EPLs was proposed by Simon, Glick, and Frank in 1972. It categorizes lesions according to the primary source of infection and the interaction between pulpal and periodontal disease, thereby guiding appropriate treatment planning [4].

The 2017 World Workshop classification provides a contemporary consensus-based framework for EPLs, distinguishing lesions of endodontic or periodontal origin, combined lesions, and lesions associated with root damage. In patients with periodontitis, lesion severity is further graded according to the extent and morphology of periodontal destruction, facilitating standardized diagnosis and treatment planning [5].

The Foce classification represents a simplified clinical framework for categorizing EPLs according to the main pathways of interaction between periodontal and endodontic infections. Owing to its simplicity and suitability for clinical research, it was selected as the primary classification system in the present study. It distinguishes Class 1 (crown-down periodontal lesions), Class 2 (down-crown lesions of endodontic origin), Class 3 (combined endo-periodontal lesions), and a pseudo-class comprising conditions that mimic endo-periodontal lesions without true pulpal–periodontal communication. Although not included in the official 2017 World Workshop classification, it represents a practical tool for clinical and research purposes [6].

Since EPLs frequently develop in the presence of periodontal destruction, understanding the classification of periodontitis is essential. The 2018 European Federation of Periodontology (EFP) classification characterizes periodontitis using a staging and grading framework. Staging reflects disease severity and treatment complexity, whereas grading estimates the rate of disease progression and considers biological and systemic risk factors. Together, these parameters provide a comprehensive assessment of periodontal disease and support individualized treatment planning [5].

Recent epidemiological evidence has demonstrated a strong association between advanced periodontal disease and the occurrence of EPLs. In particular, Sari et al. reported a prevalence of endo-periodontal lesions of 9.62%, with EPLs being most frequently observed in patients with Stage III periodontitis. Furthermore, Grade 3 EPLs and mandibular molars were the most commonly affected categories [7]. These findings suggest that increased periodontal destruction enhances anatomical communication between periodontal and pulpal tissues, facilitating lesion progression. Similarly, Ruetters et al. have confirmed that Stage III and IV periodontitis are significantly associated with a higher prevalence of EPLs, likely due to extensive attachment loss, deep periodontal pockets, and furcation involvement [8]. Furthermore, rapidly progressing periodontal disease (Grade C) has been linked to more aggressive tissue destruction, potentially increasing the likelihood of combined lesions [9].

Tooth-specific factors also play a crucial role in EPL development. Molars, particularly mandibular ones, are more frequently affected due to complex root anatomy, the presence of furcation areas, and a higher density of accessory canals [10]. Additional systemic and behavioral factors, including aging, smoking, diabetes mellitus, and poor oral hygiene, have also been identified as potential contributors to EPL occurrence and severity [11].

Despite growing evidence, limited data exist regarding the combined influence of periodontal stage and grade on the distribution and subtype of EPLs in large clinical populations. In particular, few studies have simultaneously evaluated Stage I–IV and Grade A–C classifications in relation to EPL subtypes in retrospective cohorts.

To address this knowledge gap, the present study evaluated the distribution of endo-periodontal lesion subtypes according to the Foce classification and investigated their association with periodontal stage and grade based on the current European Federation of Periodontology (EFP) classification in a retrospective cohort of patients treated at a university periodontal clinic. Unlike previous studies that primarily reported the prevalence of endo-periodontal lesions, the present study also explored the relationship between lesion subtype and periodontal stage and grade, providing additional evidence that may improve the clinical characterization and assessment of these complex lesions. Therefore, the aim of this study was to evaluate the clinical and statistical distribution of endo-periodontal lesions and investigate their association with periodontal stage and grade.

## 2. Materials and Methods

### 2.1. Study Design and Setting

A retrospective observational study was conducted over a one-year period in the Department of Periodontology, Faculty of Dental Medicine, Carol Davila University of Medicine and Pharmacy, Bucharest, Romania.

The study was approved by the Ethics Committee of Carol Davila University of Medicine and Pharmacy, Bucharest, Romania (Protocol No. 17996, approved on 25 June 2026) and conducted in accordance with the Declaration of Helsinki.

### 2.2. Study Population

The initial study population comprised 884 patients who were examined and treated in the Department of Periodontology over a one-year period. Among these, 31 patients fulfilled the diagnostic criteria for endo-periodontal lesions according to the Foce classification system and were subsequently included in the dedicated analysis of this subgroup.

### 2.3. Case Identification and Diagnostic Assessment

Patients diagnosed with endo-periodontal lesions were identified through review of clinical records and radiographic examinations using standardized diagnostic criteria. The diagnosis was established based on both endodontic and periodontal findings. Endodontic involvement was assessed using a cold pulp vitality test and percussion testing. Periodontal examination included probing pocket depth (PPD) measurements at six sites per tooth, clinical attachment level (CAL), bleeding on probing (BOP), tooth mobility, and furcation involvement. Radiographic assessment was performed using periapical radiographs and panoramic radiographs. The diagnosis and classification of endo-periodontal lesions were established based on the combined clinical and radiographic findings according to the Foce classification system. For patients presenting endo-periodontal lesions, additional demographic and clinical information was subsequently extracted from their medical charts and standardized clinical documentation.

### 2.4. Classification Systems

Periodontal status was classified according to the European Federation of Periodontology (EFP) framework, using stage and grade assessment. Endo-periodontal lesions were categorized according to the Foce classification system, assigning a lesion type for each case based on the combined clinical and radiographic findings.

### 2.5. Data Collection

For all patients included in the endo-periodontal lesion subgroup (*n* = 31), the following variables were recorded:

- Gender (female/male);
- Age at the time of evaluation (years);
- Type of affected tooth, categorized as monoradicular or pluriradicular;
- Associated systemic pathology (comorbidities reported by the patient and/or documented in available medical records);
- Periodontal diagnosis (EFP stage and grade);
- Endo-periodontal lesion type (Foce).

Due to the retrospective design of the study, information regarding smoking status, history of dental trauma, previous periodontal or endodontic treatment, antibiotic use, pregnancy status, and extensive dental restorations was not consistently available in the medical records and, therefore, could not be included in the analysis.

### 2.6. Inclusion and Exclusion Criteria

Patients diagnosed with periodontitis according to the EFP classification, who had available clinical and radiographic examinations, were screened for eligibility. Those presenting endo-periodontal lesions diagnosed according to the Foce classification system, based on combined clinical and radiographic findings, were included in the study.

Patients without endo-periodontal lesions or with incomplete clinical or radiographic documentation preventing definitive diagnosis and classification were excluded from the analysis.

### 2.7. Examiner Assessment

The clinical and radiographic data analyzed in this retrospective study were obtained from patient records completed by seven clinicians during routine clinical practice. Each patient was evaluated by a single clinician as part of routine care. However, the identification and selection of eligible cases were performed through a manual review of all patient records by a single investigator. Patients with periodontitis were screened using the available clinical and radiographic data, and the diagnosis of endo-periodontal lesions according to the Foce classification was established consistently by the same investigator for all included cases. Owing to the retrospective nature of the study, no predefined examiner calibration protocol had been implemented; therefore, inter-examiner agreement and formal reliability analysis could not be assessed.

### 2.8. Data Analysis

All data were analyzed using IBM SPSS Statistics for Windows, Version 25.0 (IBM Corp., Armonk, NY, USA), and Microsoft Excel and Word 2024 (Microsoft Corporation, Redmond, WA, USA). Quantitative variables were tested for normal distribution using the Shapiro–Wilk test and are reported as averages with standard deviations or medians with interquartile ranges. Qualitative variables are reported as counts or percentages.

Quantitative variables with non-parametric distribution were tested between EPL groups using the Mann–Whitney U test. Measures of associations between EPL classification and other qualitative variables were assessed using Fisher’s exact test. Prediction of having EPL 3 was carried out using univariable and multivariable binomial logistic regression models using EFP grading (while also adjusting for age and gender). Models were tested for significance and goodness-of-fit. Performance of the prediction was analyzed using odds ratios with 95% confidence intervals along with significance values. The threshold considered for the significance level for all tests was considered to be α = 0.05.

## 3. Results

Table 1 summarizes the characteristics of the analyzed patients. Results show that the distribution according to gender is almost equal, with a slight predominance of women (51.6%), and the mean age was 53.55 ± 9.42 years (median = 52 years). According to EFP staging, most of the patients had Stage III (54.8%) and Grade B (71%). EPL classification shows that most of the patients had EPL 3 lesions (58.1%). A total of 16 patients (41.9%) presented with comorbidities; 12 patients (38.7%) had hypertension; 3 patients (9.67%) had diabetes mellitus; and 1 patient (3.2%) had asthma. According to affected teeth, most of them were pluriradicular (64.5%).

**Figure 1:**
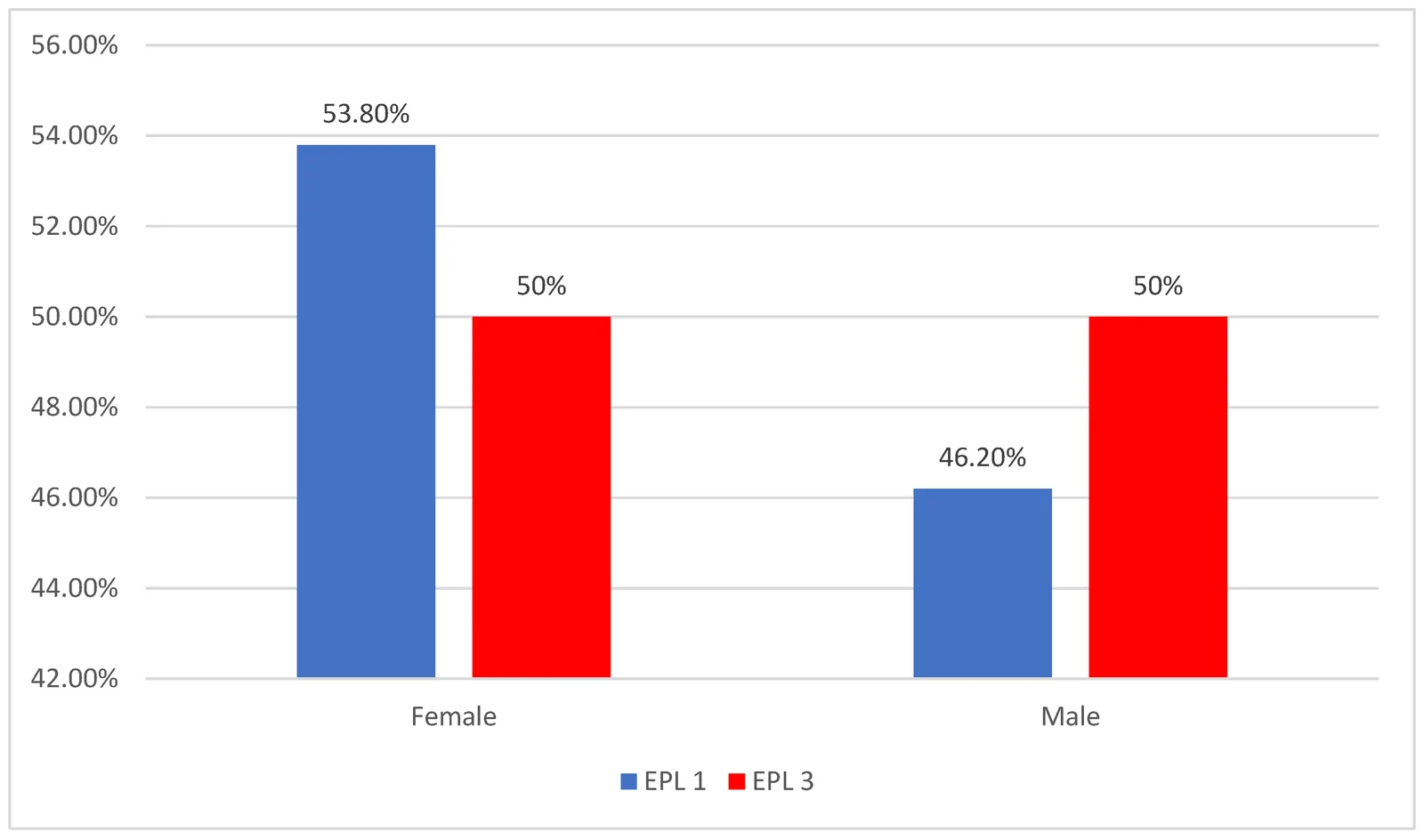
Distribution of endo-periodontal lesion (EPL) types according to gender. No significant differences were observed between male and female patients.

**Figure 2:**
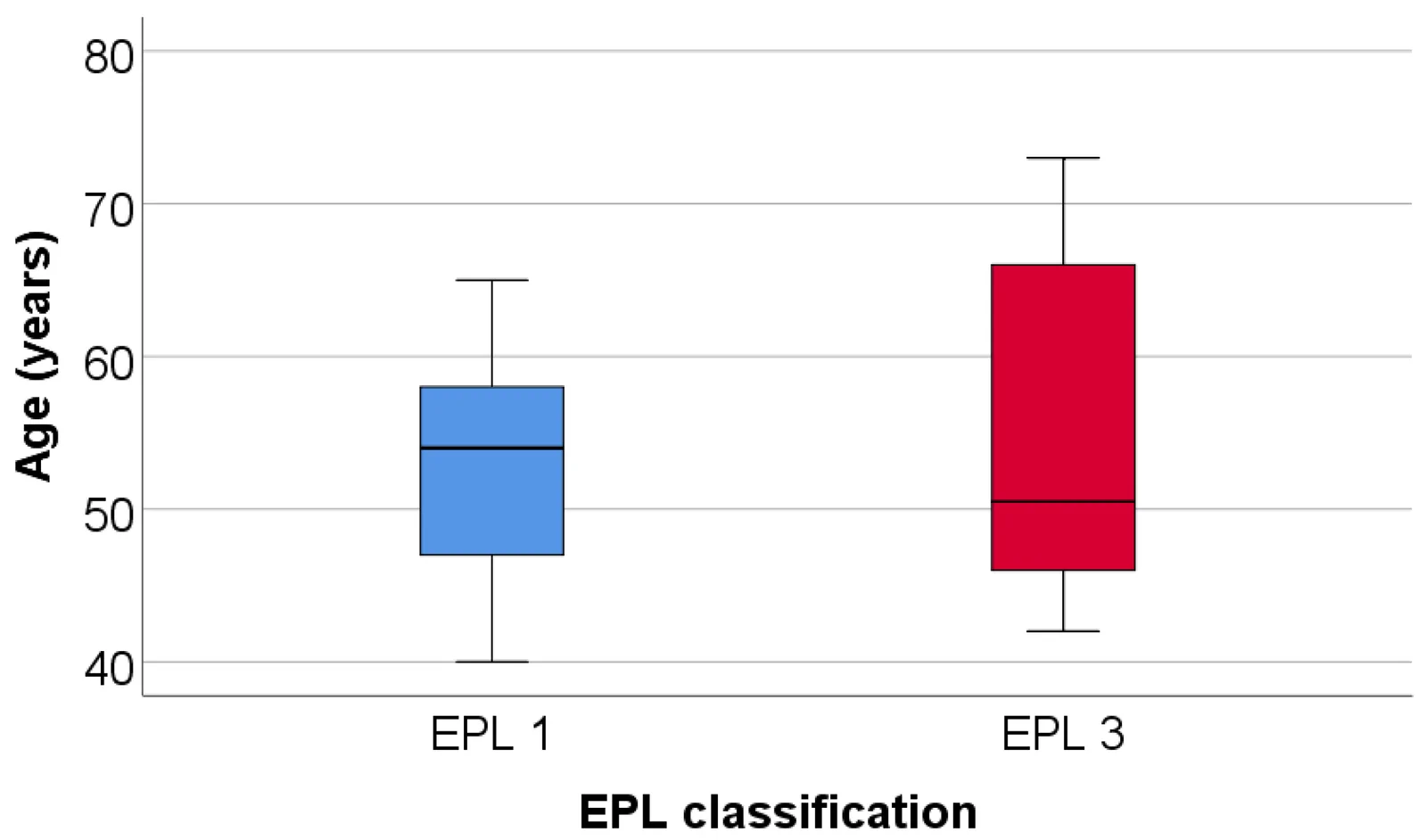
Boxplot showing the distribution of patients’ age according to endo-periodontal lesion (EPL) type. Although patients with EPL 1 appeared to have a slightly higher median age, the age distributions largely overlapped, and no statistically significant difference was observed between the two groups.

**Figure 3:**
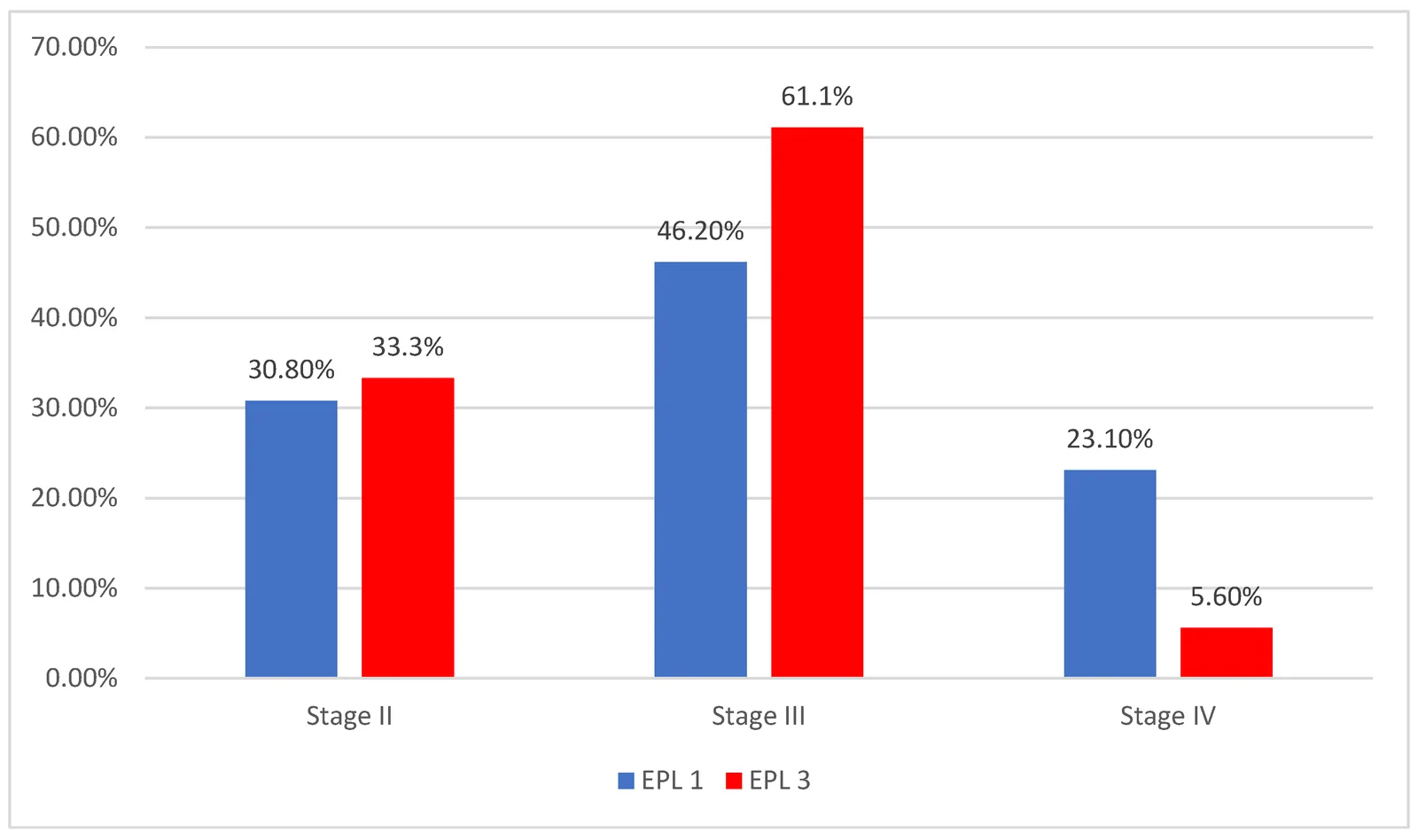
Distribution of patients according to EFP stage and endo-periodontal lesion (EPL) type. EPL 3 was most frequently observed in patients with Stage III periodontitis (61.1%), whereas Stage IV periodontitis was more frequent among patients with EPL 1 (23.1% vs. 5.6%).

**Figure 4:**
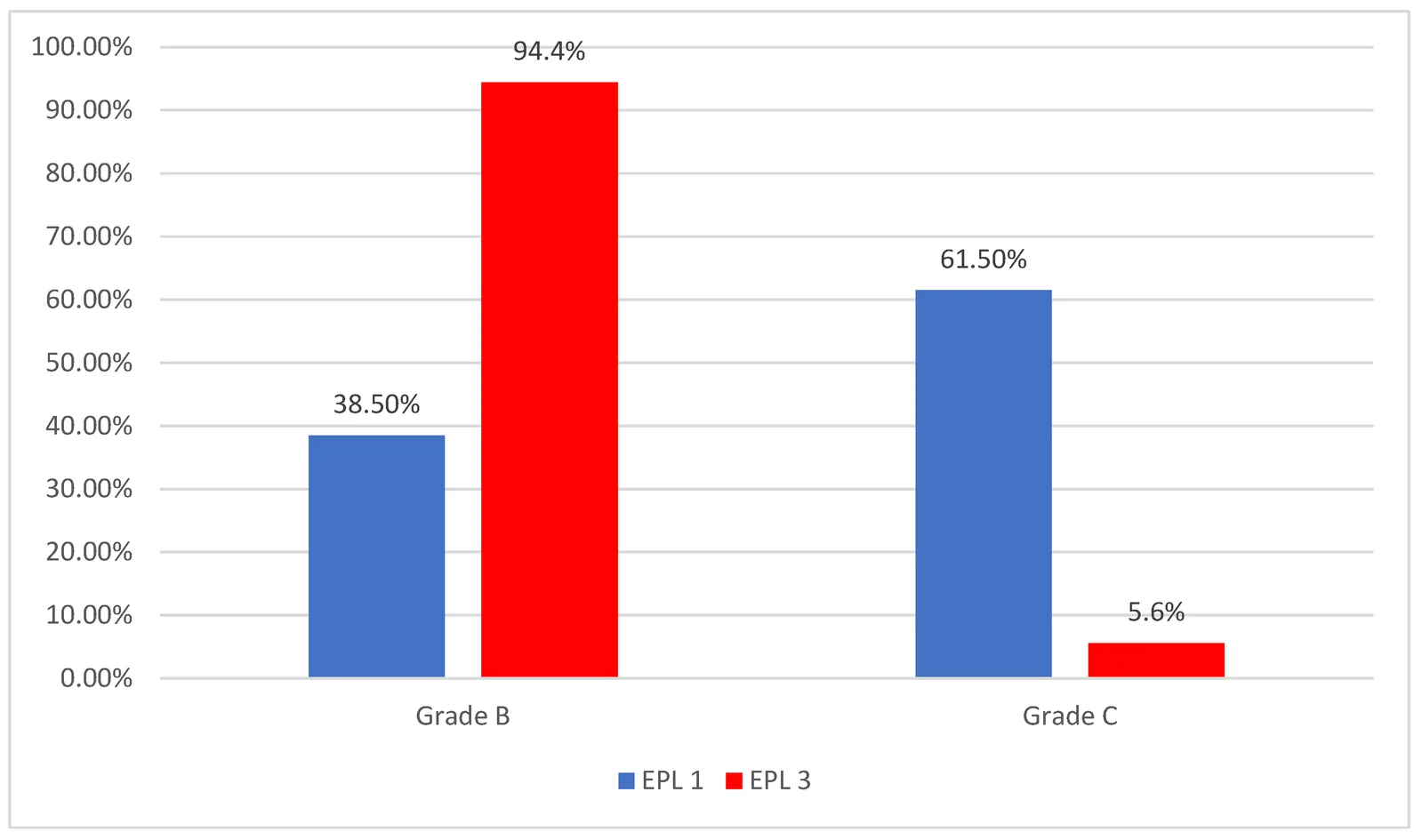
Distribution of patients according to EFP grade and endo-periodontal lesion (EPL) type. Patients with EPL 3 were predominantly classified as EFP Grade B (94.4%), whereas EFP Grade C was more frequently observed among patients with EPL 1 (61.5%). This distribution was statistically significant (*p* = 0.001).

**Figure 5:**
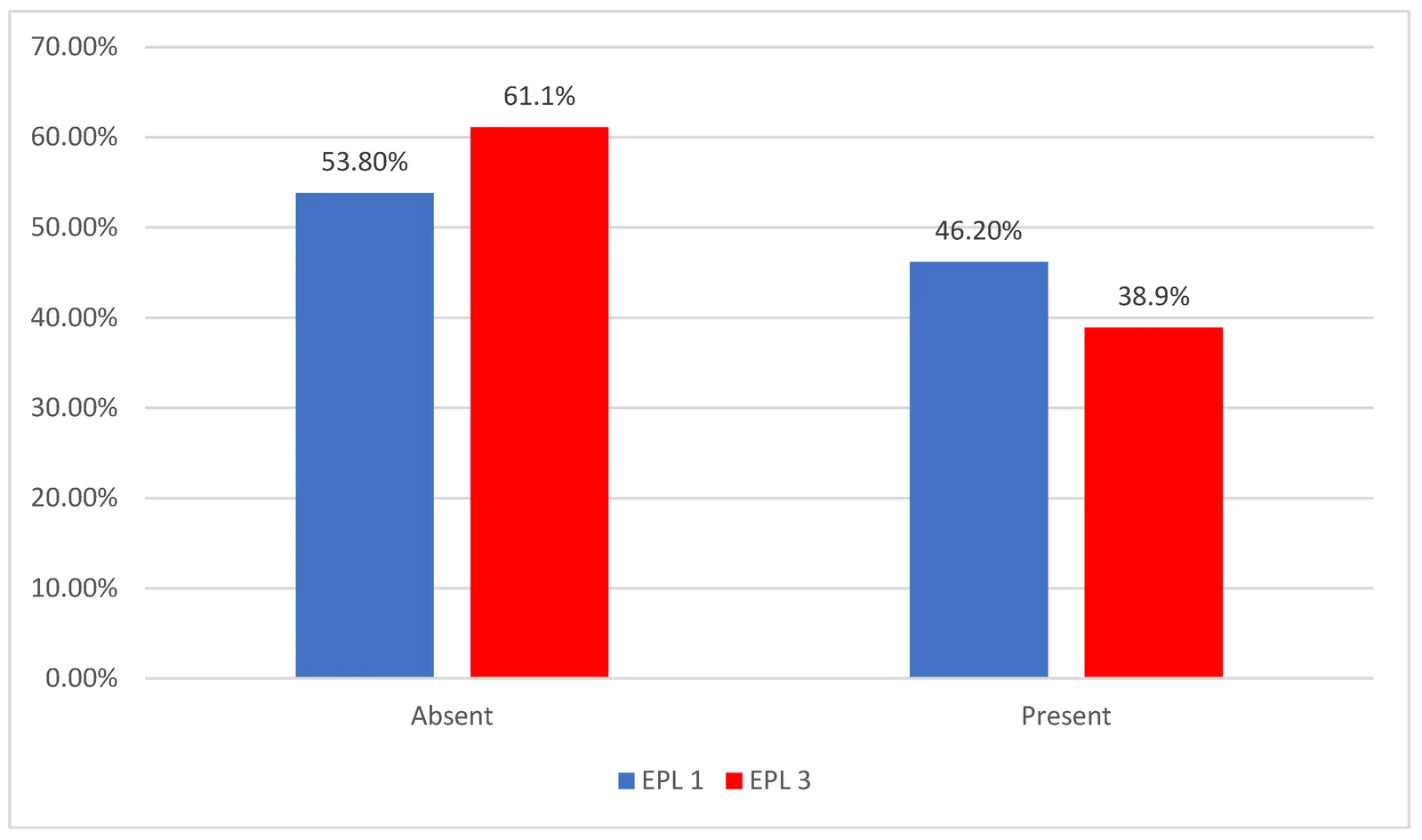
Distribution of patients according to the presence of comorbidities and endo-periodontal lesion (EPL) type. Patients without comorbidities represented the majority in both groups, with a slightly higher proportion among those with EPL 3. No statistically significant association between the presence of comorbidities and EPL type was observed.

**Figure 6:**
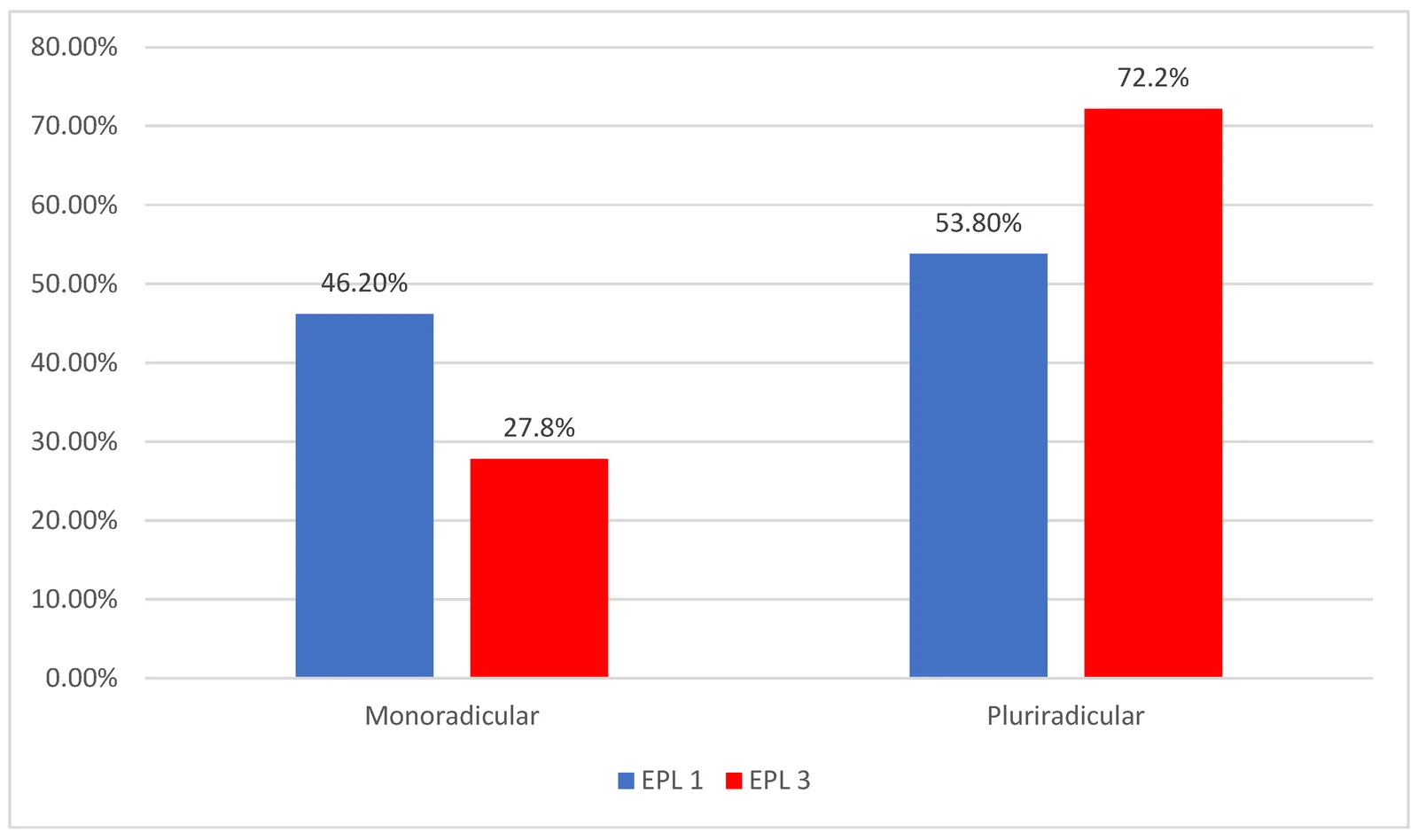
Distribution of patients according to tooth type and endo-periodontal lesion (EPL) type. Pluriradicular teeth were more frequently affected than monoradicular teeth in both EPL groups, particularly among patients with EPL 3. However, no statistically significant association was observed between tooth type and EPL classification.

Data from Table 2, Figure 1, Figure 2, Figure 3, Figure 4, Figure 5 and Figure 6 and Supplementary Materials illustrate the comparison of analyzed parameters according to EPL classification. Results show that the only significant differences observed according to EPL classification were in EFP grading (*p* = 0.001), where patients with EFP Grade B were significantly more associated with EPL 3 than EPL 1 (94.4% vs. 38.5%), while patients with EFP Grade C were significantly more associated with EPL 1 than EPL 3 (61.5% vs. 5.6%).

Prediction of EPL 3 in a univariable binomial logistic regression model using EFP grading shows that patients with Grade B had increased odds of having EPL 3 in comparison to patients with Grade C by 27.2 times (OR 95% CI = 2.712–272.828). This effect remains significant while adjusting for age and gender in a multivariable binomial logistic regression model (EFP Grade B—OR = 32.676, 95% CI = 2.832–376.984; male gender—OR = 0.584, 95% CI = 0.087–3.915, *p* = 0.580; age—OR = 0.993, 95% CI = 0.904–1.092, *p* = 0.892).

Statistically significant differences were observed only in relation to EFP grading. Patients with EFP grade B were significantly more associated with EPL 3 lesions compared to EPL 1 (94.4% vs. 38.5%, *p* = 0.001). Conversely, patients with EFP Grade C were significantly more frequently associated with EPL 1 than EPL 3 (61.5% vs. 5.6%, *p* = 0.001).

In the univariable binomial logistic regression model, EFP Grade B was strongly associated with EPL 3, showing 27.2 times higher odds compared to Grade C (OR = 27.2; 95% CI: 2.712–272.828). This association remained significant after adjustment for age and gender in the multivariable model, where Grade B patients had 32.676 times higher odds of presenting EPL 3 (OR = 32.676; 95% CI: 2.832–376.984).

Neither gender nor age showed a statistically significant influence on EPL 3 occurrence (male gender: OR = 0.584, *p* = 0.580; age: OR = 0.993, *p* = 0.892).

**Figure 7:**
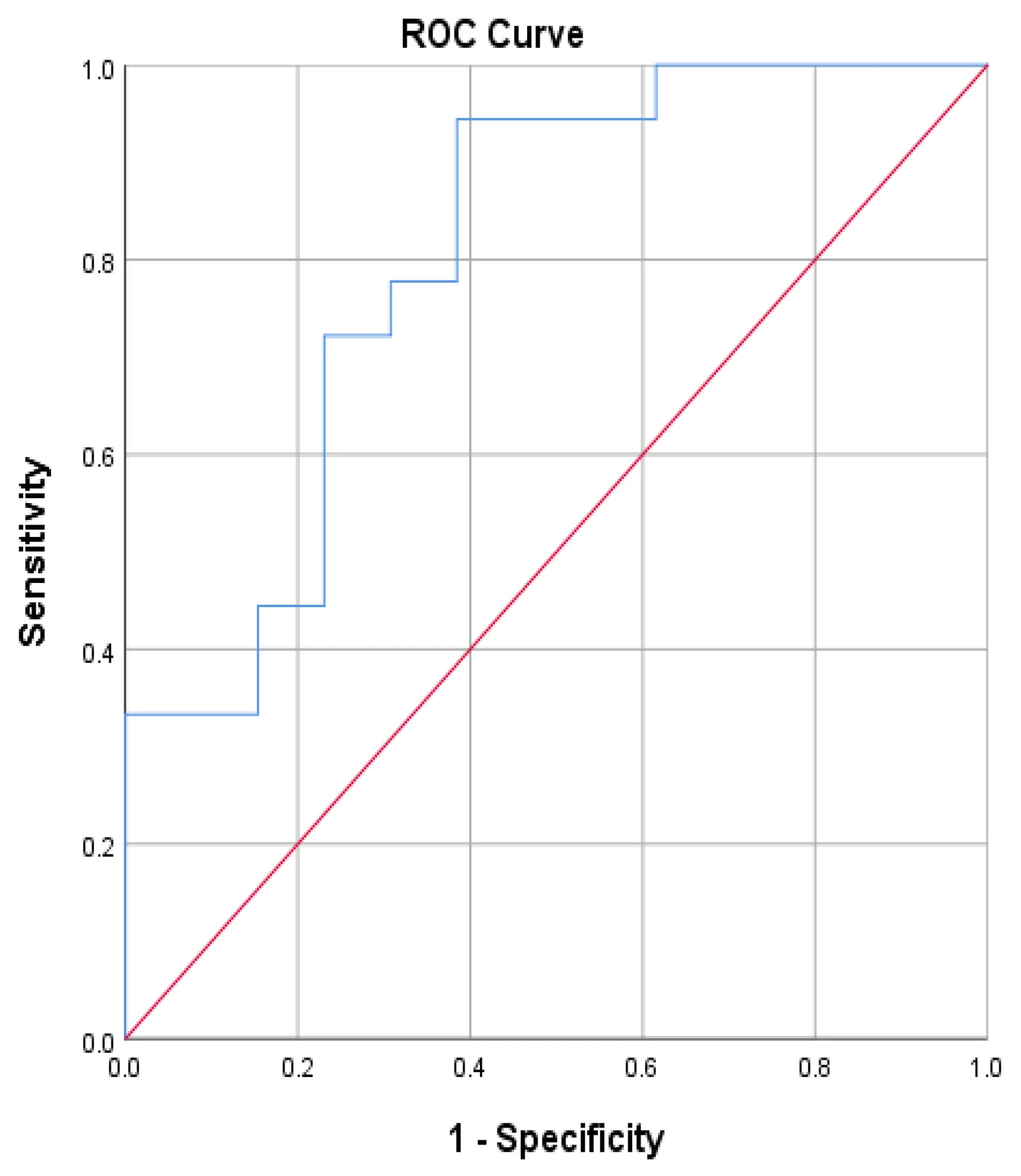
Receiver operating characteristic (ROC) curve of the multivariable logistic regression model predicting EPL 3. The blue line represents the ROC curve, while the red diagonal line represents the reference line corresponding to no discriminatory ability. The model demonstrated good discriminative performance, with an area under the curve (AUC) of 0.803 (95% CI: 0.641–0.966), indicating good ability to distinguish between EPL 3 and EPL 1.

The adjusted model demonstrated acceptable calibration (Hosmer–Lemeshow *p* = 0.398), a Nagelkerke R^2^ of 0.451, an overall classification accuracy of 80.6%, and good discriminatory ability (AUC = 0.803, 95% CI 0.641–0.966) (Figure 7).

## 4. Discussion

The present study demonstrates an association between EFP grading and the distribution of EPLs, with EFP Grade B showing a markedly higher likelihood of association with EPL 3, even after adjustment for age and gender. This finding suggests that periodontal disease progression, as captured by the EFP grading system, may be a more influential determinant of EPL complexity than demographic factors, reinforcing the importance of biologically driven classification frameworks in endo-periodontal diagnostics.

When interpreted in the context of existing literature, our findings further highlight the substantial heterogeneity in reported EPL prevalence and distribution. Ruetters et al. reported a low prevalence of EPLs (4.9% at patient level and 0.4% at tooth level) in a university hospital cohort using the 2017 classification system, reflecting a more conservative diagnostic environment with strict inclusion criteria [8].

In contrast, Güneç et al., using panoramic radiographic assessment, reported a substantially higher prevalence of 31.6%, suggesting that imaging modality and diagnostic sensitivity strongly influence EPL detection rates [12].

In a more clinically selected periodontitis population, Sari et al. reported a prevalence of 9.62%, indicating that EPLs are relatively frequent within periodontal cohorts but remain highly dependent on case definition and classification adherence [7].

Similarly, Dako et al. reported a 4% prevalence based on periapical radiographic assessment and additionally highlighted smoking as a potential modifying factor, supporting the role of systemic and behavioral risk indicators in endo-periodontal pathology [13].

Grudianov et al. reported a higher prevalence of 17.78% in periodontitis patients, further emphasizing the variability introduced by diagnostic criteria and study design [14].

Collectively, these studies underscore that EPL prevalence is highly inconsistent across the literature, ranging from approximately 4% to over 30%, largely due to heterogeneity in population selection, imaging techniques, and diagnostic thresholds. In this context, the primary contribution of the present study is not prevalence estimation but rather the identification of a statistical association between EFP grading and EPL type distribution, particularly the strong predictive value of EFP Grade B for EPL 3.

The finding that EPL 3 was significantly associated with intermediate periodontal severity (Grade B), rather than the most advanced stage (Grade C), is clinically relevant and may appear counterintuitive. This could suggest that true combined endo-periodontal lesions are more likely to develop in teeth where both periodontal and endodontic pathways remain biologically active and capable of interaction, whereas in advanced periodontal destruction (Grade C), lesions may be predominantly periodontal in origin with less structural or biological interplay between compartments.

A total of 16 patients (41.9%) presented with comorbidities, while 12 patients (38.7%) had hypertension, 3 patients (9.67%) had diabetes mellitus, and 1 patient (3.2%) had asthma. Among these conditions, diabetes mellitus and hypertension may be particularly relevant to the periodontal component of endo-periodontal lesions, given their established associations with periodontal inflammation and disease progression [15,16].

Although Grade B periodontitis emerged as an independent predictor of EPL 3 in our cohort, this finding should be interpreted with caution. The low number of Grade C patients presenting EPL 3 may be partly explained by the limited sample size, resulting in unstable estimates and wide confidence intervals. Therefore, the observed association should be considered exploratory rather than conclusive.

An alternative explanation is that teeth affected by rapidly progressing Grade C periodontitis may be extracted at earlier stages because of extensive periodontal destruction, thereby reducing the likelihood of observing mature combined endo-periodontal lesions. However, this hypothesis could not be evaluated within the present retrospective study and requires confirmation in larger prospective investigations.

Furthermore, the absence of significant effects of age and gender reinforces the hypothesis that EPL development is primarily driven by local pathological processes rather than systemic demographic determinants. This supports the concept that EPLs represent a biologically complex entity governed by the interaction between pulpal infection, periodontal biofilm dynamics, and anatomical communication pathways, rather than patient-level characteristics.

From a clinical standpoint, these findings emphasize that reliance on severity staging alone is insufficient for EPL characterization. Instead, accurate diagnosis requires an integrated endodontic–periodontal approach combining clinical examination, radiographic interpretation, and structured classification systems. The strong association between EFP Grade B and EPL 3 further suggests that intermediate periodontal destruction may represent a critical window for the development of true combined lesions, with direct implications for early diagnosis and treatment planning.

The present study has several limitations that should be acknowledged. First, the relatively small sample size may have reduced the statistical power and stability of the multivariable logistic regression model, increasing the potential risk of model overfitting and resulting in wide confidence intervals for some estimates. Nevertheless, endo-periodontal lesions are relatively uncommon and can be difficult to identify.

Consequently, the estimated odds ratios should be interpreted with caution, as they reflect considerable uncertainty regarding the magnitude of the observed associations. Although the multivariable analysis was limited to clinically relevant variables (periodontal grade, age, and gender), the small number of outcome events should be considered when interpreting the adjusted estimates. Nevertheless, the adjusted model demonstrated acceptable calibration and discriminatory ability, supporting its use as an exploratory analytical approach. Furthermore, the retrospective single-center design limited the analysis to the information available in the patients’ medical records and may have introduced referral bias, as patients treated in a university periodontal clinic may not be representative of the general population. As a result, potentially important confounding variables, including smoking status, history of dental trauma, extensive dental restorations, and previous endodontic treatment, were not consistently recorded and could not be incorporated into the statistical analysis.

In addition, microbiological analyses were not available, and the retrospective design precluded long-term follow-up of the included patients.

Finally, the clinical and radiographic data were derived from examinations performed by seven different clinicians during routine clinical practice without a predefined calibration protocol. Consequently, inter-examiner agreement could not be assessed, as a formal reliability analysis (e.g., Cohen’s kappa) was not performed.

These limitations should be considered when interpreting the present findings. Given the relatively small sample size, the present study may be considered a pilot study, and its findings require confirmation in larger prospective multicenter studies with standardized examiner calibration, comprehensive data collection, microbiological assessment, and long-term follow-up.

## 5. Conclusions

Within the limitations of this retrospective study, EPL 3 was the most frequently encountered EPL subtype and was predominantly associated with pluriradicular teeth. A significant association was identified between EFP grading and EPL subtype distribution, with Grade B patients showing substantially higher odds of presenting EPL 3 compared to Grade C patients. Age and gender did not significantly influence lesion distribution, suggesting that local disease-related factors may play a greater role in EPL development than demographic characteristics.

Further prospective multicenter studies including larger and more representative patient cohorts are required to validate these findings and clarify the biological mechanisms underlying the relationship between periodontal disease progression and EPL subtype development. Future investigations should also incorporate standardized examiner calibration, comprehensive assessment of relevant clinical confounders (including smoking status, previous endodontic treatment, dental trauma, and extensive restorations), microbiological analyses, and long-term follow-up to provide a more robust understanding of endo-periodontal lesion pathogenesis and progression.

## Figures and Tables

**Table 1:** Demographic and clinical characteristics of patients with endo-periodontal lesions included in the study.

Parameter	Value
Gender n (%) (Female)	16 (51.6%)
Age (Mean ± SD) (Median (IQR))	53.55 ± 9.42, 52 (47–60)
EFP stage n (%)	
Stage II	10 (32.3%)
Stage III	17 (54.8%)
Stage IV	4 (12.9%)
EFP grading n (%)	
Grade B	22 (71%)
Grade C	9 (29%)
EPL n (%)	
EPL 1	13 (41.9%)
EPL 3	18 (58.1%)
Comorbidities n (%)	13 (41.9%)
Tooth type n (%)	
Monoradicular	11 (35.5%)
Pluriradicular	20 (64.5%)

EFP—European Federation of Periodontology; EPL—endo-periodontal lesion.

**Table 2:** Comparison of analyzed parameters according to EPL classification. * Fisher’s exact test; ** Mann–Whitney U test.

Parameter	EPL 1	EPL 3	*p*
Gender n (%)			
Female	7 (53.8%)	9 (50%)	1.000 *
Male	6 (46.2%)	9 (50%)	
Age (Median (IQR))	54 (47–59)	50.5 (45.75–66.25)	0.921 **
EFP stage n (%)			
Stage II	4 (30.8%)	6 (33.3%)	0.455 *
Stage III	6 (46.2%)	11 (61.1%)	
Stage IV	3 (23.1%)	1 (5.6%)	
EFP grade n (%)			
Grade B	5 (38.5%)	17 (94.4%)	0.001 *
Grade C	8 (61.5%)	1 (5.6%)	
Comorbidities n (%)			
Absent	7 (53.8%)	11 (61.1%)	0.727 *
Present	6 (46.2%)	7 (38.9%)	
Tooth type n (%)			
Monoradicular	6 (46.2%)	5 (27.8%)	0.449 *
Pluriradicular	7 (53.8%)	13 (72.2%)	

EFP—European Federation of Periodontology; EPL—endo-periodontal lesion.

## Data Availability

The data supporting the findings of this study are available from the corresponding author upon reasonable request. The data are not publicly available due to ethical and privacy restrictions involving patient information.

## Supplementary Materials

The following supporting information can be downloaded at: https://www.mdpi.com/article/10.3390/healthcare14162551/s1, Supplementary Statistical Analysis.

## Abbreviations

The following abbreviations are used in this manuscript:

EPL	Endo-periodontal lesion
EPLs	Endo-periodontal lesions
EFP	European Federation of Periodontology
ROC	Receiver operating characteristic
