# Supplementary material for: Periodontal Disease and Endo-Periodontal Lesion Subtypes: A Retrospective Study
Source: Healthcare (Basel). 2026 Aug 14;14(16):2551. doi: 10.3390/healthcare14162551 (PMC13512781; doi:10.3390/healthcare14162551)
Supplement: Supplementary file 1 [file healthcare-14-02551-s001.zip › healthcare-4446222-supplementary.pdf]

First model—dependent variable: EPL; independent variable: EFP grading

Case Processing Summary

Unweighted Cases[a]		N	Percent
Selected Cases	Included in Analysis	31	100.0
	Missing Cases	0	0.0
	Total	31	100.0
Unselected Cases		0	0.0
Total		31	100.0

a. If weight is in effect, see classification table for the total number of cases.

Dependent Variable Encoding

Original Value	Internal Value
EPL 1	0
EPL 3	1

Categorical Variables Codings

		Frequency	Parameter coding (1)
EFP grading	Grade B	22	0.000

	Grade C	9	1.000

Classification Table[a,b]

Observed			Predicted		
			EPL		Percentage Correct
			EPL 1	EPL 3	
Step 0	EPL	EPL 1	0	13	0.0
		EPL 3	0	18	100.0
	Overall Percentage				58.1

a. Constant is included in the model.

b. The cut value is 0.500

Variables in the Equation

		B	S.E.	Wald	df	Sig.	Exp(B)
Step 0	Constant	0.325	0.364	0.799	1	0.371	1.385

Variables not in the Equation

			Score	df	Sig.
Step 0	Variables	EFP grading(1)	11.482	1	0.001
	Overall Statistics		11.482	1	0.001

Block 1: Method = Enter

Omnibus Tests of Model Coefficients

		Chi-square	df	Sig.
Step 1	Step	12.304	1	0.000
	Block	12.304	1	0.000
	Model	12.304	1	0.000

Model Summary

Step	-2 Log likelihood	Cox & Snell R Square	Nagelkerke R Square
1	29.861[a]	0.328	0.441

a. Estimation terminated at iteration number 5 because parameter estimates changed by less than 0.001.

Hosmer and Lemeshow Test

Step	Chi-square	df	Sig.
1	0.000	0	.

Contingency Table for Hosmer and Lemeshow Test

		EPL = EPL 1		EPL = EPL 3		
		Observed	Expected	Observed	Expected	Total
Step 1	1	8	8.000	1	1.000	9
	2	5	5.000	17	17.000	22

Classification Table[a]

Observed			Predicted		
			EPL		Percentage Correct
			EPL 1	EPL 3	
Step 1	EPL	EPL 1	8	5	61.5
		EPL 3	1	17	94.4
	Overall Percentage				80.6

a. The cut value is 0.500

Variables in the Equation

		B	S.E.	Wald	df	Sig.	Exp(B)	95% C.I.for EXP(B)	
								Lower	Upper
Step 1[a]	EFP grading(1)	-3.303	1.176	7.885	1	0.005	0.037	0.004	0.369
	Constant	1.224	0.509	5.786	1	0.016	3.400		

a. Variable(s) entered on step 1: EFP grading.

Casewise List[b]

Case	Selected Status[a]	Observed EPL	Predicted	Predicted Group	Temporary Variable Resid	ZResid	SResid
24	S	3**	0.111	1	0.889	2.828	2.223

a. S = selected, U = unselected cases, and ** = misclassified cases.

b. Cases with studentized residuals greater than 2.000 are listed.

Probability saved by the model (PRE_1) is verified in the ROC curve below.

```
ROC PRE_1 BY EPL (3)
  /PLOT=CURVE(REFERENCE)
  /PRINT=SE COORDINATES
  /CRITERIA=CUTOFF(INCLUDE) TESTPOS(LARGE) DISTRIBUTION(FREE) CI(95)
  /MISSING=EXCLUDE.
```

ROC Curve

Case Processing Summary

EPL	Valid N (listwise)

Positive[a]	18
Negative	13

Larger values of the test result variable(s) indicate stronger evidence for a positive actual state.

a. The positive actual state is EPL 3.

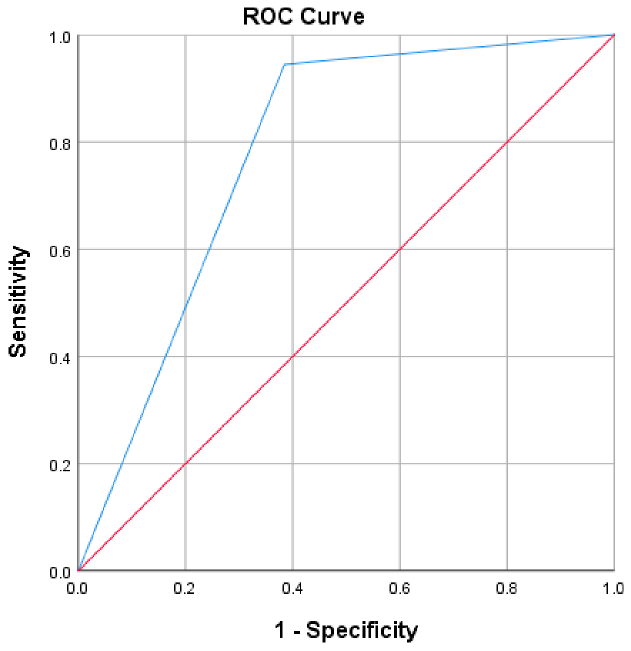

Diagonal segments are produced by ties.

Area Under the Curve

Test Result Variable(s): Predicted probability

Area	Std. Error[a]	Asymptotic Sig.[b]	Asymptotic 95% Confidence Interval	
			Lower Bound	Upper Bound
0.780	0.092	0.009	0.599	0.960

The test result variable(s): Predicted probability has at least one tie between the positive actual state group and the negative actual state group. Statistics may be biased.

a. Under the nonparametric assumption

b. Null hypothesis: true area = 0.5

Coordinates of the Curve

Test Result Variable(s): Predicted probability

Positive if Greater Than or Equal To[a]	Sensitivity	1 - Specificity
0.0000000	1.000	1.000
0.4419192	0.944	0.385
1.0000000	0.000	0.000

The test result variable(s): Predicted probability has at least one tie between the positive actual state group and the negative actual state group.

a. The smallest cutoff value is the minimum observed test value minus 1, and the largest cutoff value is the maximum observed test value plus 1. All the other cutoff values are the averages of two consecutive ordered observed test values.

Second model—dependent variable: EPL; independent variables: EFP grading, age, gender (female—reference).

Case Processing Summary

Unweighted Cases[a]		N	Percent
Selected Cases	Included in Analysis	31	100.0
	Missing Cases	0	0.0
	Total	31	100.0
Unselected Cases		0	0.0
Total		31	100.0

a. If weight is in effect, see classification table for the total number of cases.

Dependent Variable Encoding

Original Value	Internal Value
EPL 1	0
EPL 3	1

Categorical Variable Codings

		Frequency	Parameter coding (1)
Gender	Female	16	0.000
	Male	15	1.000
EFP grading	Grade B	22	0.000
	Grade C	9	1.000

Classification Table[a,b]

Observed			Predicted EPL: EPL 1	Predicted EPL: EPL 3	Percentage Correct
Step 0	EPL	EPL 1	0	13	0.0
		EPL 3	0	18	100.0
	Overall Percentage				58.1

a. Constant is included in the model.

b. The cut value is 0.500

Variables in the Equation

	B	S.E.	Wald	df	Sig.	Exp(B)

Step 0	Constant	0.325	0.364	0.799	1	0.371	1.385

Variables not in the Equation

			Score	df	Sig.
Step 0	Variables	EFP grading(1)	11.482	1	0.001
		Age (years)	0.266	1	0.606
		Gender(1)	0.045	1	0.833
	Overall Statistics		11.701	3	0.008

Block 1: Method = Enter

Omnibus Tests of Model Coefficients

		Chi-square	df	Sig.
Step 1	Step	12.647	3	0.005
	Block	12.647	3	0.005
	Model	12.647	3	0.005

Model Summary

Step	-2 Log likelihood	Cox & Snell R Square	Nagelkerke R Square

1	29.518[a]	0.335	0.451

a. Estimation terminated at iteration number 5 because parameter estimates changed by less than 0.001.

Hosmer and Lemeshow Test

Step	Chi-square	df	Sig.
1	8.368	8	0.398

Contingency Table for Hosmer and Lemeshow Test

		EPL = EPL 1		EPL = EPL 3		
		Observed	Expected	Observed	Expected	Total
Step 1	1	3	2.760	0	0.240	3
	2	2	2.631	1	0.369	3
	3	3	2.609	0	0.391	3
	4	0	0.866	3	2.134	3
	5	2	0.824	1	2.176	3
	6	0	0.786	3	2.214	3
	7	1	0.763	2	2.237	3
	8	1	0.567	2	2.433	3
	9	1	0.524	2	2.476	3
	10	0	0.669	4	3.331	4

Classification Table[a]

Observed			Predicted EPL: EPL 1	Predicted EPL: EPL 3	Percentage Correct
Step 1	EPL	EPL 1	8	5	61.5
		EPL 3	1	17	94.4
	Overall Percentage				80.6

a. The cut value is 0.500

Variables in the Equation

		B	S.E.	Wald	df	Sig.	Exp(B)	95% C.I.for EXP(B)	
								Lower	Upper
Step 1[a]	EFP grading(1)	-3.487	1.248	7.808	1	0.005	0.031	0.003	0.353
	Age (years)	-0.007	0.048	0.018	1	0.892	0.993	0.904	1.092
	Gender(1)	-0.537	0.970	0.306	1	0.580	0.584	0.087	3.915
	Constant	1.895	2.740	0.478	1	0.489	6.653		

a. Variable(s) entered on step 1: EFP grading, age (years), gender.

Casewise List[b]

Case	Selected Status[a]	Observed EPL	Predicted	Predicted Group	Temporary Variable: Resid	Temporary Variable: ZResid	Temporary Variable: SResid

24	S	3**	0.126	1	0.874	2.634	2.185

a. S = selected, U = unselected cases, and ** = misclassified cases.

b. Cases with studentized residuals greater than 2.000 are listed.

Probability saved by the model (PRE_2) is verified in the ROC curve below.

```
ROC PRE_2 BY EPL (3)
  /PLOT=CURVE(REFERENCE)
  /PRINT=SE COORDINATES
  /CRITERIA=CUTOFF(INCLUDE) TESTPOS(LARGE) DISTRIBUTION(FREE) CI(95)
  /MISSING=EXCLUDE.
```

Case Processing Summary

EPL	Valid N (listwise)
Positive[a]	18
Negative	13

Larger values of the test result variable(s) indicate stronger evidence for a positive actual state.

a. The positive actual state is EPL 3.

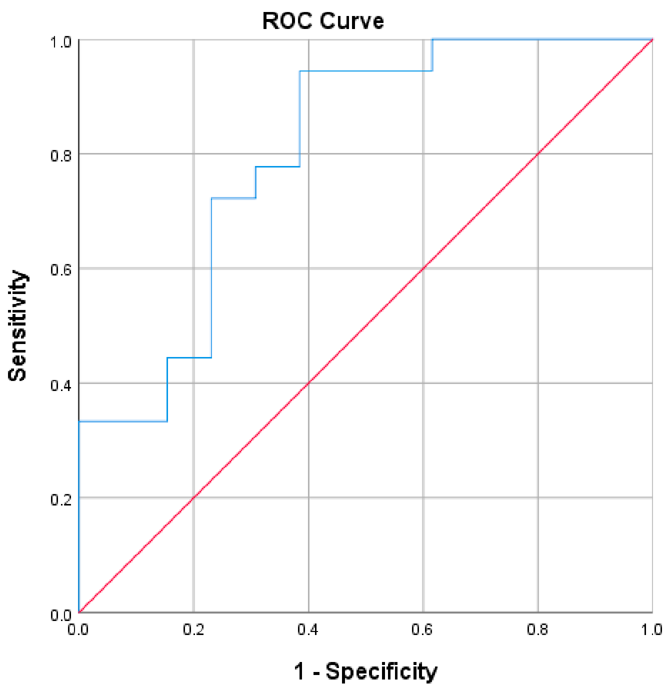

Area Under the Curve

Test Result Variable(s): Predicted probability

Area	Std. Error[a]	Asymptotic Sig.[b]	Asymptotic 95% Confidence Interval	
			Lower Bound	Upper Bound
0.803	0.083	0.004	0.641	0.966

a. Under the nonparametric assumption

b. Null hypothesis: true area = 0.5

Coordinates of the Curve

Test Result Variable(s): Predicted probability

Positive if Greater Than or Equal To[a]	Sensitivity	1 - Specificity
0.0000000	1.000	1.000
0.0788574	1.000	0.923
0.0812456	1.000	0.846
0.1014911	1.000	0.769
0.1217035	1.000	0.692
0.1241739	1.000	0.615
0.1281327	0.944	0.615
0.4187466	0.944	0.385
0.7091924	0.889	0.385
0.7132111	0.833	0.385
0.7178541	0.778	0.385
0.7224576	0.778	0.308
0.7276505	0.722	0.308
0.7315222	0.722	0.231
0.7365914	0.667	0.231
0.7416451	0.556	0.231
0.7435137	0.500	0.231
0.7472213	0.444	0.231
0.7784462	0.444	0.154
0.8097195	0.389	0.154

0.8127279	0.333	0.154
0.8176005	0.333	0.077
0.8238695	0.333	0.000
0.8271610	0.278	0.000
0.8294821	0.222	0.000
0.8313192	0.167	0.000
0.8326881	0.111	0.000
0.8340452	0.056	0.000
1.0000000	0.000	0.000

a. The smallest cutoff value is the minimum observed test value minus 1, and the largest cutoff value is the maximum observed test value plus 1. All the other cutoff values are the averages of two consecutive ordered observed test values.
